# Comparison of the Performance of Iranian Azeri-Speaking Children Based on Iran and Reference Bayley III Norms

**DOI:** 10.22037/ijcn.v16i2.32930

**Published:** 2022-03-14

**Authors:** Nahideh HASANI KHIABANI, Mohammad BARZEGAR, Sina RAEISI, Marzieh JALALIAN CHALESHTORI, Seifollah HEIDARABADI, Ali BAHARI GHAREHGOZ

**Affiliations:** 1 Pediatric Health Research Center, Tabriz University of Medical Science, Tabriz, Iran; 2 Department of Speech Therapy, Faculty of Rehabilitation, Tabriz University of Medical Science, Tabriz, Iran; 3 Department of Educational Sciences, Farhangian University, Tehran Iran

**Keywords:** Bayley III Scales, Cognitive, Iranian Children, Bayley III Norms

## Abstract

**Objectives:**

The present study aimed to compare the performance of Iranian Azeri-speaking children based on Iran and the reference of the Bayley Scales of Infant and Toddler Development Third Edition (Bayley III) norms.

**Materials & Methods:**

The total sample included 248 infants and toddlers aged 16 days to 42 months and 15 days. The Iranian version of the Bayley III scale was used in this study. The scaled scores and composite scores in cognitive, language, and motor domains were compared based on Iran and reference Bayley III norms. Then, the proportions of children scoring < -1 standard deviation (SD) and < -2 SD were compared based on the two norms.

**Results:**

The scaled scores of the study group were higher based on the reference norms in receptive communication, fine motor, and gross motor subtests. The scaled scores were variable in expressive communication and cognitive subtests. The differences were significant for receptive communication and fine motor subtests (P<0.05). Using the reference norms instead of Iran norms resulted in under-referral regarding receptive communication and fine motor subtests. More children scored below 1 and 2 SD using Iran norms in comparison to those reported for using the reference norms.

**Conclusion:**

Iran norms differ significantly from the reference norms over two subscales. It is recommended to use population-specific norms to identify children with developmental delay and early intervention.

## Introduction

Annually, approximately 7.6 million children under the age of 5 die in developing countries, and about 200 million children under the age of 5 do not reach their full potential in these countries. These countries lose almost 20% of their adult productivity (1, 2). In the US, about 13% of children under 15 years have a kind of developmental disability, and about 1.6% of children have global developmental delay (3). In Iranian children, the prevalence of global delay is about 14.6% (4). 

In developing countries, most children with mild to moderate developmental problems are identified late and generally in preschool ages (5). However, studies support the effectiveness of early intervention in children with developmental problems (6). Early detection and early intervention improve a child’s functional abilities and help acquire critical functional skills. Nevertheless, without an appropriate and reliable screening and diagnostic tool, the detection of children with abnormal development might be difficult (7). 

There are numerous forms of developmental assessment, and it is necessary to determine which type of test is suitable. The Bayley Scales of Infant and Toddler Development, known as a gold standard tool, evaluates infants’ and toddlers’ developmental status in five key developmental domains, including cognition, language, motor, social-emotional, and adaptive behavior at the age of under 42 months. The third edition of this scale (i.e., Bayley III) was published in 2006 (8). The Bayley III was designed and normed in the US. It seems that the results of the US norms might be probably invalid when it is used in other populations not designed for because several factors, such as family financial condition, geographic location, ethnicity, religious, iodine and iron deficiencies, inadequate cognitive and social-emotional stimulation, and maternal depression, can affect all domains of child development (9).

If the US cut-off points are at a lower level than the children’s developmental situation area, it would cause a group of children who need intervention to miss the chance of early intervention. However, if the US cut-off points were at a higher level than the children’s developmental situation area, it would lead to unnecessary referrals and an increase in parental concerns and extra costs. In this regard, some countries, such as South Africa, Sri Lanka, Australia, Netherlands, Brazil, and others, have evaluated the validity of the Bayley III for their populations and compared children’s mean standardized scores to reference Bayley III norms. There are some differences between the results of these comparisons (10-14).

The Bayley III scale has been developed and validated by the Ministry of Health and Medical Educational of Iran in cooperation with the University of Social Welfare and Rehabilitation Sciences of Tehran, Iran (15-17). Since only Iranian Persian-speaking children participated in the adaptation and validation of the Bayley III scale in Iran, the present study aimed to compare the performance of Iranian Azeri-speaking children in the motor, language, and cognitive domains of the Bayley III according to Iran and the reference norms. 

## Materials & Methods

The present cross-sectional study was conducted on a total of 248 infants and toddlers under 42 months of age selected from the clients of the Tabriz Growth and Development Center, East Azerbaijan, Iran. The sample was an available sample of children who met inclusion criteria. The inclusion criteria consisted of children within 16 days to 42 months and 15 days of age, birth weight of at least 2500 g, and gestational age of at least 36 weeks. The exclusion criteria included known developmental disabilities (including autism spectrum disorder and cerebral palsy), moderate to severe developmental delay, and sensory disabilities (e.g., blindness and deafness). The study group was divided into seven age groups. The number of children in each age group was according to the ratio of each age group to the total number. In the study group, 53% and 47% of the participants were male and female, respectively. The demographic data were gathered from the parents of children prior to the administration of the test (Table 1).

This study used the Iranian version of the Bayley III scale. The Bayley III is an individually administered instrument that measures the developmental level of children within 16 days to 42 months and 15 days of age. This tool consists of five subtests, namely cognitive, receptive communication, expressive communication, fine motor, and gross motor (18, 19). A few modifications were made to the Iranian version of Bayley III to increase its application in Iranian culture, especially in the subtests of receptive and expressive communications and in the illustration book (15). 

The Bayley III in Persian-speaking children is a reliable and valid instrument with good psychometric characteristics in Iranian children. In the Persian version of the Bayley III, the Cronbach’s alpha coefficient in all domains was above 0.74; Pearson correlation coefficient in various domains was ≥ 0.982 in the test-retest method and ≥ 0.993 in the inter-rater method indicating very good internal consistency between the items of the subscales (16). The reliability coefficients of the original Bayley were within the range of 0.67-0.94, with correlations increasing as age increased (20, 21). All the five subtests of the Iranian version of the Bayley III (i.e., cognitive, receptive communication, expressive communication, fine motor, and gross motor) were administered according to the instruction by an experienced psychologist. Depending on the child’s age, it took 50-90 minutes to administer the test. All participants underwent the test at the same testing center. 

Raw scores were calculated for five subtests. They were converted into scaled scores, composite scores, and percentile ranks using the tables in both the Iran and reference norms of the Bayley III scale. The scaled scores have been scaled within a range of 1-19, a mean of 10, and a standard deviation of 3. The composite scores have been scaled within a range of 40-160, a mean of 100, and a standard deviation of 15 (22).

The Ethics Committee of Tabriz University of Medical Sciences approved this study. Written informed consent was obtained from the parents.

**Data Analysis**

The data were analyzed by SPSS software (version 16.0; SPSS, Inc., Chicago, IL, US). Initially, the variables were statistically checked for normality by a one-sample Kolmogorov-Smirnov test. The normally distributed data were shown as mean±standard deviation and compared by an independent samples t-test. Nonparametric variables were shown as median with interquartile range and compared using the Mann-Whitney U-test between the study groups. A p-value of less than 0.05 was considered statistically significant.

## Results

The obtained results revealed that the mean standardized scores were different based on the reference and Iran norms in the total sample (n=248). The mean scaled scores of the study group were higher based on the reference norms in receptive communication, fine motor, and gross motor subtests. The differences were statistically significant for receptive communication and fine motor subtests (P<0.05). There were no statistically significant differences in the mean scaled scores of cognitive, expressive communication, and gross motor subtests. The mean composite scores of the motor domain were significantly different (Table 2). The smallest mean difference was observed for the cognitive subtest for the age group four (i.e., 18 months 16 days to 24 months 15 days). The largest mean difference was observed for receptive communication for age group one (i.e., 16 days to 6 months 15 days) ( 3-9).

For the cognitive subtest, the largest differences between the reference and Iran norms were observed in children within 12 months 16 days to 18 months 15 days of age. For the receptive communication subtest, the largest differences were observed in children within 16 days to 6 months 15 days of age. Regarding the expressive communication subtest, the largest differences were observed in children within 6 months 16 days to 12 months 15 days of age. Regarding the fine motor subtest, the largest differences were observed in children within 18 months 16 days to 24 months 15 days of age. For the gross motor subtest, the largest differences between the reference and Iran norms were observed in children within 18 months 16 days to 24 months 15 days of age. Regarding the composite scores, the largest differences between the reference and Iran norms were observed in the language domain in children within 16 days to 6 months 15 days of age and motor domain in children within 18 months 16 days to 24 months 15 days of age.

By means of a scaled score of 7 (-1 standard deviation [SD]) or 4 (-2 SD) as the cut-off point, the results showed that for all subtests, different proportions of children with low scores were identified using Iran and reference norms in the total sample. When using the reference norms instead of Iran norms, fewer children scored below 1 or 2 SD in all subtests in the total sample. The results varied in different age groups (Table 10).

## Discussion

The current study performed on 248 children under 3 years of age in Tabriz aimed to develop psychomotor profiles and compare scores based on Iran and reference norms on the Bayley III scale. The results showed differences between the study group scores based on Iran and reference norms. The study group scored higher based on reference norms in receptive communication, fine motor, and gross motor subtests. The mean scaled scores were significantly different in the receptive communication and fine motor subtests. The differences were not statistically significant in the cognitive, expressive communication, and gross motor subtests. The composite scores were different based on Iran and reference norms in all domains except the cognitive domain. The differences were significant in the motor domain and were not significant in the language domain. 

With regard to the age groups, the standardized scores in the age groups two (i.e., 6 months 16 days to 12 months 15 days), three (12 months 16 days to 18 months 15 days), and five (24 months 16 days to 30 months 15 days) were not significantly different between Iran and reference norms. The mean scaled scores of the receptive communication in the age groups one (i.e., 16 days to 6 months 15 days) and seven (i.e., 36 months 16 days to 42 months 15 days) were significantly higher based on the reference norms. In age group four (i.e., 18 months 16 days to 24 months 15 days), the mean scaled scores of fine motor and gross motor subtests were significantly higher based on reference norms. In age group six (i.e., 30 months 16 days to 36 months 15 days), the mean scaled scores of the gross motor subtest were significantly higher based on the reference norms. 

The reasons for the above-mentioned differences are not entirely clear. An important explanation might be the differences between the reference and Iran norms samples. The reference sample included a clinical group (10% of the normative sample), children with syndromes and developmental disorders. These children would have scored low on the Bayley III scale, lowering their mean values. The Iranian children sample comprised only healthy children, which might be the reason for the higher mean scores of the Iranian children sample than the reference norms. Other possible explanations for these results include demographic, cultural, and socioeconomic differences between the structures of the two populations (23). Furthermore, previous studies have shown that Iranian children scored higher than US children in the Ages & Stages Questionnaire (24). 

Another factor affecting development is maternal education level. In the US, educational level was measured in years, and 42%, 30%, and 28% of mothers had low, medium, and high levels of education, respectively (25). In the present study, 22.5%, 61.2%, and 16.3% of mothers had low, medium, and high levels of education, respectively. The differences might be explained by differences in child-rearing practices. Iranian children might have more exposure to mother-child interaction and be more encouraged in physical practice early in life, developing their performances (26).

In relation to the findings of the current study regarding the receptive communication, fine motor, and gross motor subtests, under-referral would have resulted from the reference norms. In other words, the use of reference norms would have overestimated a child’s functional ability in comparison to Iran norms. These results are in accordance with the results of previous studies and have shown that the Bayley-III scale seriously underestimates developmental delay in other populations (27).

In a study conducted by Steenis et al. in the Netherlands, 1912 children were assessed by the Bayley III scale. The results revealed that using the reference norms leads to over-referral in the gross motor subtest and under-referral in the cognitive, expressive communication, and fine motor subtests (10). Similar to the present study, using the reference norms leads to the misclassification of the developmental delay category.

In a study performed by Chinta et al. in Australia, 156 three-year-old children were assessed using the Bayley III scale. The results revealed that their scores were higher in the cognitive, fine motor, receptive communication, and expressive communication subtests (14). Similar to the current study, using the reference norms leads to the under-referral of high-risk children.

In another study conducted by Rademeyer et al. on black South Africans, 122 children aged 2-13 months were assessed using the Bayley III scale. The results demonstrated that black African urban infants perform better than American infants on the Bayley III scale (13). Similar to the present study results, the researchers concluded that using the reference norms leads to the overestimation of black African children’s performance. 

In a study carried out by Godamunne et al. in Sri Lanka, 150 children with 6, 12, and 24 months of age were assessed by the Bayley III scale. The results revealed that at 12 months, the Sri Lankan children scored significantly higher than the US children in the cognitive subtest but lower in the gross motor subtest. In contrast, at 24 months, the US children’s scores were higher than the Sri Lankan children’s scores on the cognitive subtest, and there was no significant difference between their scores on the motor subtest (12). Similar to the current study results, using the reference norms instead of specific norms leads to the misclassification of high-risk children.

**Table 1.** Socio-demographic information of the participant children

		**Total**
**Total N**		248
**Mean age (month)**		20
**Boys **		131
**Girls**		117
**Mean gestational age in weeks**		37+4
**Mean birth weight(g)**		3020
**Mother** ** 's** **education level %**	Low* Moderate* High*	22.5 61.2 16.3
**N per age group**		
**1** **: 16 days-6 months 15 days**		14
**2** **: 6 months 16 days- 12 months 15 days**		46
**3** **: 12 months 16 days- 18 months 15 days**		73
**4** **: 18 months 16 days- 24 months 15 days**		46
**5** **: 24 months 16 days-30 months 15 days**		27
**6** **: 30 months 16 days-36 months 15 days**		24
**7** **: 36 months 16 days – 42 months 15 days**		18

* Low education level: under Diploma Degree

*Moderate education level: Diploma until Bachelor Degree

*High education level: Master Degree and more

**Table 2:** The results on Bayley III scale for all age groups

**Bayley Scales**	**IRAN (n=248)**	**USA (n=248)**	**Mann-Whitney U-test value**	**p-value**
Cognition	8 (6-10)	8 (7-10)	30370	0.808^a^
Receptive Communication	8 (6-10)	9 (8-11)	26730	0.011^a,^*
Expressive Communication	8.12 ± 3.92	8.10 ± 3.22	-	0.950^b^
Fine Motor	7 (5-9.75)	8 (7-10)	26000	0.003^a,^*
Gross Motor	8 (5-10)	9 (6-10)	28030	0.086^a^
Composite Cognition	90 (80-100)	90 (85-100)	30330	0.789^a^
Composite Language	90 (79-103)	91 (83.75-103)	28824	0.226^a^
Composite Motor	86.5 (76-97)	91 (82-100)	26092	0.003^ a,^*
Percentile Cognition	25 (9-50)	25 (16-50)	30330	0.789^a^
Percentile Language	25 (8-58)	27 (14.25-58)	28830	0.227^a^
Percentile Motor	18.5 (5-42)	27 (12-50)	26080	0.003^a,^*

^a^Data are shown as median with interquartile range and were compared by Mann-Whitney U-test.^b^Data are shown as mean ± standard deviation and were compared by independent samples t-test. *Statistically significant (p<0.05).

**Table 3:** The results on Bayley III scale for infants less than 6 months of age

**Bayley Scales**	**IRAN (n=14)**	**USA (n=14)**	**p-value**
Cognition	8.50 ± 1.99	8.43 ± 2.21	0.929
Receptive Communication	9.50 ± 1.22	12.07 ± 1.14	<0.001*
Expressive Communication	9.71 ± 1.54	9.14 ± 1.41	0.315
Fine Motor	9.43 ± 2.71	9.29 ± 2.64	0.889
Gross Motor	10.79 ± 1.97	10.00 ± 1.96	0.300
Composite Cognition	92.50 ± 9.95	92.14 ± 11.04	0.929
Composite Language	97.29 ± 5.06	103.64 ± 4.58	0.002*
Composite Motor	100.93 ± 11.35	98.00 ± 11.02	0.495
Percentile Cognition	34.57 ± 16.61	34.53 ± 16.71	0.995
Percentile Language	43.07 ± 13.02	59.43 ± 11.87	0.002*
Percentile Motor	52.64 ± 25.09	45.43 ± 24.08	0.445

Data are shown as mean ± standard deviation. Means were compared by independent samples t-test. *Statistically significant (p<0.05

**Table 4:** The results on Bayley III scale for children 6-12 months of age

**Bayley Scales**	**IRAN (n=46)**	**USA (n=46)**	**Mann-Whitney U-test value**	**p-value**
Cognition	8.72 ± 3.43	9.02 ± 2.39	-	0.623^a^
Receptive Communication	9.11 ± 2.85	8.85 ± 2.53	-	0.644^a^
Expressive Communication	10.26 ± 3.06	9.07 ± 2.71	-	0.050^a^
Fine Motor	7.57 ± 3.35	7.85 ± 2.56	-	0.650^a^
Gross Motor	9 (4-11)	9 (3-10)	974	0.509^b^
Composite Cognition	93.59 ± 17.15	95.11 ± 11.95	-	0.623^a^
Composite Language	98.17 ± 14.71	94.09 ± 12.90	-	0.160^a^
Composite Motor	86.02 ± 18.73	85.35 ± 16.19	-	0.854^a^
Percentile Cognition	41.01 ± 29.25	40.87 ± 23.48	-	0.980^a^
Percentile Language	46.41 ± 28.40	38.20 ± 24.62	-	0.142^a^
Percentile Motor	29.56 ± 25.69	26.28 ± 22.30	-	0.515^a^

^a^Data are shown as mean ± standard deviation and were compared by independent samples t-test. ^b^Data are shown as median with interquartile range and were compared by Mann-Whitney U-test.*Statistically significant (p<0.05).

**Table 5:** The results on Bayley III scale for children 12-18 months of age

**Bayley Scales**	**IRAN (n=73)**	**USA (n=73)**	**Mann-Whitney U-test value**	**p-value**
Cognition	7 (5-10)	9 (6-10)	2363.5	0.236^a^
Receptive Communication	8 (7-10)	9 (7-10)	2382	0.265^a^
Expressive Communication	7.88 ± 3.54	7.73 ± 2.89	-	0.779^b^
Fine Motor	7.04 ± 3.13	7.81 ± 2.46	-	0.102^b^
Gross Motor	7.00 ± 3.57	7.15 ± 3.50	-	0.797^b^
Composite Cognition	85 (75-100)	95 (80-100)	2364.5	0.237^a^
Composite Language	88.78 ± 16.08	88.82 ± 16.19	-	0.988^b^
Composite Motor	82.15 ± 16.89	84.89 ± 15.31	-	0.306^b^
Percentile Cognition	16 (5-50)	37 (9-50)	2364.5	0.236^a^
Percentile Language	23 (6-54)	27 (8-46)	2536.5	0.616^a^
Percentile Motor	16 (4-34)	16 (6.5-34)	2429.5	0.356^a^

^a^Data are shown as median with interquartile range and were compared by Mann-Whitney U-test. ^b^Data are shown as mean ± standard deviation and were compared by independent samples t-test. *Statistically significant (p<0.05).

**Table 6:** The results on Bayley III scale for children 18-24 months of age

**Bayley Scales**	**IRAN (n=46)**	**USA (n=46)**	**Mann-Whitney U-test value**	**p-value**
Cognition	7.59 ± 4.19	7.52 ± 3.03	-	0.932^a^
Receptive Communication	8.91 ± 4.47	9.30 ± 3.79	-	0.652^a^
Expressive Communication	7.57 ± 4.01	7.39 ± 3.68	-	0.829^a^
Fine Motor	7 (5-9)	9 (8-10)	688	0.004^b,^*
Gross Motor	7 (3-9)	9 (6.75-10)	756.5	0.018^b,^*
Composite Cognition	87.93 ± 20.94	87.61 ± 15.16	-	0.932^a^
Composite Language	89.96 ± 22.82	90.59 ± 20.17	-	0.889^a^
Composite Motor	85 (74.5-91.75)	94 (85-97)	656	0.002^b,^*
Percentile Cognition	34.59 ± 31.95	28.93 ± 23.96	-	0.340^a^
Percentile Language	35.65 ± 33.87	35.35 ± 30.40	-	0.965^a^
Percentile Motor	20.55 ± 21.41	33.82 ± 23.00	-	0.005^a,^*

^a^Data are shown as mean ± standard deviation and were compared by independent samples t-test. ^b^Data are shown as median with interquartile range and were compared by Mann-Whitney U-test.*Statistically significant (p<0.05).

**Table 7:** The results on Bayley III scale for children 24-30 months of age

**Bayley Scales**	**IRAN (n=27)**	**USA (n=27)**	**Mann-Whitney U-test value**	**p-value**
Cognition	7.00 ± 3.64	6.85 ± 2.71	-	0.866^a^
Receptive Communication	8 (5-9)	9 (8-10)	296.5	0.234^b^
Expressive Communication	5.56 ± 3.97	6.74 ± 3.53	-	0.252^a^
Fine Motor	6.41 ± 2.99	7.11 ± 2.65	-	0.364^a^
Gross Motor	6.89 ± 4.49	8.26 ± 4.71	-	0.279^a^
Composite Cognition	84.81 ± 18.11	84.26 ± 13.57	-	0.899^a^
Composite Language	78.93 ± 21.42	85.19 ± 17.57	-	0.246^a^
Composite Motor	80.07 ± 20.33	86.15 ± 20.05	-	0.274^a^
Percentile Cognition	26.94 ± 25.15	21.79 ± 16.69	-	0.380^a^
Percentile Language	8 (2-34)	18 (6-34)	286	0.174^b^
Percentile Motor	22.61 ± 23.50	29.83 ± 27.82	-	0.308^a^

^a^Data are shown as mean ± standard deviation and were compared by independent samples t-test. ^b^Data are shown as median with interquartile range and were compared by Mann-Whitney U-test.*Statistically significant (p<0.05).

**Table 8:** The results on Bayley III scale for children 30-36 months of age

**Bayley Scales**	**IRAN (n=24)**	**USA (n=24)**	**p-value**
Cognition	7.67 ± 3.21	7.79 ± 2.08	0.874
Receptive Communication	8.04 ± 2.87	8.91 ± 2.08	0.233
Expressive Communication	7.96 ± 4.65	8.58 ± 3.78	0.612
Fine Motor	7.25 ± 3.45	8.41 ± 2.90	0.212
Gross Motor	8.83 ± 4.01	11.58 ± 4.46	0.030*
Composite Cognition	88.33 ± 16.06	88.96 ± 10.42	0.874
Composite Language	88.3 ± 19.62	92.88 ± 15.32	0.376
Composite Motor	88.37 ± 19.45	100.12 ± 19.36	0.041*
Percentile Cognition	30.55 ± 27.22	2.42 ± 15.71	0.629
Percentile Language	31.84 ± 31.65	36.58 ± 28.30	0.587
Percentile Motor	33.61 ± 29.87	51.67 ± 32.36	0.050

Data are shown as mean ± standard deviation. Means were compared by independent samples t-test. *Statistically significant (p<0.05).

**Table 9.** The results on Bayley III scale for children 36-42 months of age

**Bayley Scales**	**IRAN (n=18)**	**USA (n=18)**	**p-value**
Cognition	8.33 ± 3.24	8.11 ± 1.71	0.799
Receptive Communication	7.00 ± 3.71	9.39 ± 2.17	0.026*
Expressive Communication	7.83 ± 4.58	9.50 ± 3.00	0.206
Fine Motor	7.00 ± 4.31	9.28 ± 3.14	0.080
Gross Motor	8.56 ± 3.33	10.78 ± 3.42	0.056
Composite Cognition	91.67 ± 16.18	90.56 ± 8.56	0.799
Composite Language	85.00 ± 22.38	96.00 ± 13.13	0.083
Composite Motor	86.78 ± 20.30	100.33 ± 17.89	0.041*
Percentile Cognition	36.84 ± 29.20	29.33 ± 16.71	0.352
Percentile Language	28.81 ± 32.26	40.72 ± 24.27	0.220
Percentile Motor	28.96 ± 30.11	46.83 ± 29.29	0.080

Data are shown as mean ± standard deviation. Means were compared by independent samples t-test. *Statistically significant (p<0.05).

**Table 10:** Proportion of children with low scores based on the reference and Iran norms

	US norms <- 1SD%	Iran norm <- 1SD%	US norms <- 2SD%	Iran norms <- 2SD%
All age group				
Cognition	22.1	31.8	7.6	11.6
Receptive communication	12.5	24.5	5.2	8
Expressive communication	29	32.6	7.6	11.2
Fine Motor	20.5	34.2	7.2	14.5
Gross Motor	23.7	31	12.7	18.5
Age group				
A: 16 days-6 months 15 days				
Cognition	14	14	7	7
Receptive communication	----	----	-----	-----
Expressive communication	----	----	-----	-----
Fine Motor	14	14	-----	-----
Gross Motor	----	----	-----	-----
B: 6 months 16 days- 12 months 15 days				
Cognition	10	21	2.1	6.5
Receptive communication	4.3	15	4.3	4.3
Expressive communication	8.6	10	2.1	4.3
Fine Motor	15.2	32	8.6	13
Gross Motor	30	30	21	21
C: 12 months 16 days- 18 months 15 days				
Cognition	26	34	9.5	9.5
Receptive communication	13	21.9	2.7	2.7
Expressive communication	27	36.9	6.8	4.1
Fine Motor	19	39.7	5.4	13.6
Gross Motor	25	31.5	20.5	20.5
D: 18 months 16 days- 24 months 15 days				
Cognition	32.6	41.3	13	21.7
Receptive communication	19.5	28.2	10.8	15.2
Expressive communication	41.3	45.6	13	13
Fine Motor	15.2	32.6	13	15.2
Gross Motor	23.9	41.3	6.5	28.2
E: 24 months 16 days-30 months 15 days				
Cognition	33.3	29.6	11	18.5
Receptive communication	18.5	33.3	11	14.8
Expressive communication	44.4	44.4	18.5	29.6
Fine Motor	22.2	37	11	18.5
Gross Motor	25.9	33.3	18.5	18.5
F: 30 months 16 days-36 months 15 days				
Cognition	12.5	37.5	4.1	8.3
Receptive communication	8.3	25	4.1	8.3
Expressive communication	33.3	37.5	4.1	20.8
Fine Motor	25	25	4.1	16.6
Gross Motor	4.1	29.1	4.1	8.3
G: 36 months 16 days – 42 months 15 days				
Cognition	11.1	33.3	----	5.5
Receptive communication	---	55.5	-----	16.6
Expressive communication	11.1	38.8	5.5	22.2
Fine Motor	22.2	44.4	---	22.2
Gross Motor	5.5	27.7	----	5.5

## In Conclusion

Although the Bayley III scale is frequently used to assess infants’ and toddlers’ development, Iranian Azeri-speaking children performed better based on the reference norms in the present study. This finding shows the importance of population-specific norms. Given the effect of cultural and language differences on the development of children even within the same country, it is recommended to validate the Bayley III scale for different languages and cultures in Iran.

A limitation of this study is that the sample of children who were enrolled in the standardization of Bayley III in Iran did not include nonPersian-speaking children. Therefore, Iranian Azeri-speaking children were not enrolled in the standardization sample.

## Author’s Contribution

N.H.kh conceived of the presented idea. S.H developed the theoretical framework, directed and supervised the project. N.H.kh performed the examination. S. R performed the analysis of data. M.B was involved in planning the work. N.H.Kh wrote the manuscript with support from S.H. M.J contributed to writing the draft. All authors have read and approved the manuscript.

## Conflict of Interest

Not applicable
